# Scientometric analysis of highly cited papers on avascular necrosis of the femoral head from 1991 to 2022

**DOI:** 10.1186/s10195-023-00709-3

**Published:** 2023-06-15

**Authors:** Raju Vaishya, Brij Mohan Gupta, Ghouse Modin N. Mamdapur, Abhishek Vaish, Filippo Migliorini

**Affiliations:** 1 Department of Orthopaedics, Indraprastha Apollo Hospitals, 110076 New Delhi, India; 2 Council of Scientific and Industrial Research, National Institute of Science, Technology and Development Studies, New Delhi, India; 3 Department of Library and Information Science, Yenepoya University, Mangalore, Deralakatte, 575018 Karnataka India; 4 Department of Orthopedic, Trauma and Reconstructive Surgery, RWTH University Hospital of Aachen, 52064 Aachen, Germany; 5 Department of Orthopedics and Trauma Surgery, Academic Hospital of Bolzano (SABES-ASDAA), 39100 Bolzano, Italy

**Keywords:** Bibliometrics, Scientometrics, Avascular necrosis, Osteonecrosis, Femoral head, Hip

## Abstract

**Introduction:**

A highly cited paper (HCP) is considered a landmark that can influence both research and clinical practice. The characteristics of HCPs in avascular necrosis of the femoral head (AVNFH) were identified and the research status was explored in a scientometric analysis.

**Methods:**

The present bibliometric analysis were based on the Scopus database from 1991 to 2021. Microsoft Excel and VOSviewer were used for co-authorship, co-citation, and co-occurrence analysis. From 8496 papers, only 2.9% (244) were HCPs, with 200.8 citations registered per article.

**Results:**

Of the HCPs, 11.9% and 12.3% were externally funded and had international collaboration, respectively. These were published in 84 journals by 1625 authors from 425 organizations of 33 countries. The USA, Japan, Switzerland, and Israel were the leading countries.The lead research organizations were Sinai Hospital and John Hopkins University (USA). The most impactful organizations were University of Arkansas for Medical Science, and Good Samaritan Hospital (USA). R.A. Mont (USA) and K.H. Koo (South Korea) were the most prolific contributing authors, while R. Ganz (Switzerland) and R.S. Weinstein (USA) registered the most impactful contributions. The most prolific publishing journal was the *Journal of Bone and Joint Surgery*.

**Conclusion:**

The HCPs contributed to the knowledge of AVNFH by examining research perspectives and identifying important subareas through keyword analysis.

*Level of evidence*: Not applicable.

*Trial registration*: Not applicable.

## Introduction

Avascular necrosis of the femoral head (AVNFH) is an idiopathic, debilitating, and progressive disease in which local death of osteocytes and the component of the bone marrow occurs owing to venous stasis or arterial blood supply damage or interruption in the femoral head [1, 2]. The subsequent repair process attempts to heal the necrotic area, but structural deterioration and collapse of the femoral head cause pain and dysfunction of the hip joint [3, 4].

AVNFH can be classified into two major categories: traumatic and nontraumatic. Traumatic osteonecrosis is the result in 15–50% of fractures of the neck of the femur and 10–25% of hip dislocations [5]. The main causes of nontraumatic AVNFH are the use of corticosteroids, chronic alcohol overconsumption, decompression sickness, hemoglobinopathies (e.g., sickle cell disease, thalassemia, autoimmune diseases), and idiopathic causes. Smoking and obesity increase the risk of AVNFH [2, 3] and are considered to be correlated with AVNFH [2, 5]. Other less common causes and the pathophysiology of AVNFH have been described before [6–8].

An estimated 20,000 new cases of osteonecrosis are diagnosed in the USA each year, and the cumulative number of patients with AVNFH is 300,000–600,000 [9]. In recent years, approximately 12,000–24,000 new cases of osteonecrosis were diagnosed in Japan. In South Korea, the prevalence rate was 20.53 per 100,000 people in 2002; however, in 2006, this number reached 37.96, and the estimated number of new cases reached 14,103 per year on average [3]. The first large-scale epidemiological survey of nontraumatic osteonecrosis in China showed that the estimated cumulative number of patients with nontraumatic AVNFH reached 8.12 million [3].

Articles with high citations are considered central in research. Highly cited papers provide evidence and information about research trends and scientific progress in a specific field [10]. At present, a number of studies have been published in the field, including a few bibliometric studies on AVNFH [11–14]. Although the scientific publications related to AVNFH have increased during the last 30 years, their influence on biomedical literature is not known. The characteristics of highly cited papers (HCP) help us to identify important advances and their scientific impact.

Considering the clinical significance of AVNFH research and the importance of HCP, we quantitatively and qualitatively analyzed the 244 highly cited papers in this field to explore the citations, authors, journals, publishing countries, and keyword information. Furthermore, the relationships between the most frequently occurring concepts and keywords and collaboration linkages among participating countries, organizations and authors are visualized. This provides research insights into current developments from the last 30 years and will also help researchers to understand the research influences and trends, and provide a reference for future research.

## Methods

From previous bibliometric studies [15–17] on medical topics, the Scopus database was selected as the main data source for the present study, as it is the largest database for medical literature and provides the maximum number of useful metrics for a bibliometric study. A search strategy was developed to identify, retrieve, and download relevant literature in Scopus database on 30 September 2022 based on keyword tags, with the search strategy shown below. The literature search included keywords such as osteonecrosis, necrosis, femoral head, femur head, femoral head necrosis, which were placed in a Keyword tag and joined by Boolean operators, as shown in the search strategy. A time span of 30 years was set, and thus only literature published from 1991 to 2022 was included. A total of 244 records were obtained, which were ranked according to the number of citations in descending order by the sorting options in the Scopus database. Then, the citation count of minimum 100 times was chosen as a cutoff value. Finally, the top 244 records with a minimum of 100 citations obtained, which were later assessed in detailed.

The following information was collected from the 244 HPCs: publication year, citation count, funding sources, title, author, journal, country, institution, research field, and keywords. The publications data of 244 HCPs were imported from the Scopus database into Microsoft Excel (Microsoft Corp., Redmond, WA, USA), and to VOSviewer and R software for further analysis.

VOSviewer was used to visualize the collaboration network maps of countries, organizations, authors, journals, and keywords using co-authorship, co-citation, and keyword co-occurrence analysis. Specifically, a co-citation network means that two items appear together in the bibliography of a third citing item, while a co-occurrence network represents that the relationship of items is built according to the quantity of publications where they occur together. In co-authorship networks, nodes represent authors, organizations, or countries, which are connected when they share the authorship of a paper. The visualization maps mainly consist of nodes and links with different colors. Nodes in the visualization map represented the analyzed elements such as author, journal, or keyword, and the size of the nodes indicated the number of citations or occurrences The links between nodes reflected the relationship of co-citation or co-occurrence. An important parameter, total link strength (TLS), was used to quantitatively evaluate the strength of links [18].

( KEY ( osteonecrosis AND of AND femoral AND head) OR KEY ( necrosis AND of AND femoral AND head) OR KEY ( femoral AND head AND necrosis) OR KEY ( femur AND head AND necrosis) OR KEY ( necrosis AND of AND femur AND head) OR KEY ( osteonecrosis AND of AND femur AND head)) AND PUBYEAR > 1990 AND PUBYEAR > 1990.

## Results

Of the 8496 papers on AVNFH, 244 (2.87%) were HCPs and received 199–1096 citations, averaging 200.78 citations per paper (CPP). Only 29 (11.88%) of them received external funding from international and national funding agencies, and together received 5794 citations, averaging 199.79 CPP. Among the top external funding agencies supporting research in this area were the National Institute of Health, USA (12 papers); the National Institute of Arthritis & Musculoskeletal & Skin Diseases, USA; and the US Department of Health & Human Service (six papers each).

Of the 244 HCPs, 106 (43.44%) involve only one organization (i.e., zero collaborative) and together received 26,118 citations, averaging 246.39 CPP. Conversely, 138 (56.56%) HCPs were collaborative (national and international) and involve the participation of two or more organizations, and together include 22,863 citations, averaging 165.67 CPP.

Among 244 HCPs, there were 175 (71.72%) original articles, 37 (15.16%) reviews, 31 (12.70%) conference papers, and 1 (0.41%) short survey. These publications were on human subjects in 240 (98.36%) and on animal models in 17(6.97%), and some studies covered both.

Adult participation in research in this area (*n* = 110, 45.08%) accounts for the largest group of papers, followed by aged (*n* = 60, 24.59%), middle aged (*n* = 40, 16.39% share), adolescents (*n* = 22, 9.01% share), and children (*n* = 12, 4.91% share).

Clinical studies account for the largest number of papers (*n* = 128, 52.46%), followed by treatment studies (*n* = 56, 22.13%), risk factors (*n* = 24, 9.84%), epidemiology (*n* = 18, 7.38%), pathophysiology (*n* = 16, 6.56%), genetics (*n* = 3, 1.23%), and complications (*n* = 1, 0.41%).

Among the study design, controlled studies account for the largest number of papers (*n* = 85, 34.83%), followed by prospective studies (*n* = 38, 15.57%), perspective studies (*n* = 26, 10.65%), retrospective studies (*n* = 24, 9.83%), clinical trials (*n* = 20, 8.20%), controlled clinical trials (*n* = 18, 7.38%), comparative studies (*n* = 12, 4.92%), case reports (*n* = 6, 2.46%), and meta-analyses (*n* = 3, 1.22%).

Radiography was the commonest investigation modality used (*n* = 70), followed by nuclear magnetic resonance (NMR) imaging (*n* = 54), magnetic resonance imaging (MRI) (*n* = 23), computer-assisted tomography (*n* = 14), and X-ray computed tomography (*n* = 6).

### Most productive and impactful countries

Thirty-three countries were involved in the 244 HCPs, with 21 contributing 3–114 papers (Table 1), and only 4 countries contributing above the group average productivity (13.52). Only six countries registered CPP and a relative citation index (RCI) higher than the group average (177.98 and 0.89). The share of funded papers of the top 21 countries varied from 0.0% to 54.55%, with an average share of 19.01%.

**Table 1:** Bibliometric profile of the top 21 countries (with > 2 papers)

No.	Name of the country	TP	TC	CPP	RCI	ICP	%ICP	FP	%FP	TLS	Cluster number	%TP
1	USA	114	21,828	191.47	0.95	4	3.51	17	14.91	47	5	46.72
2	Japan	27	4374	162.00	0.81	5	18.52	4	14.81	5	5	11.07
3	UK	21	4098	195.14	0.97	10	47.62	6	28.57	32	3	8.61
4	South Korea	18	2442	135.67	0.68	2	11.11	1	5.56	3	4	7.38
5	France	13	2193	168.69	0.84	4	30.77	2	15.38	20	1	5.33
6	China	11	1737	157.91	0.79	4	36.36	6	54.55	6	6	4.51
7	Germany	11	1373	124.82	0.62	5	45.45	4	36.36	30	1	4.51
8	Taiwan	9	1680	186.67	0.93	3	33.33	1	11.11	12	4	3.69
9	Switzerland	8	2534	316.75	1.58	4	50.00	2	25	20	1	3.28
10	Canada	8	1403	175.38	0.87	4	50.00	3	37.5	12	2	3.28
11	Belgium	6	1106	184.33	0.92	1	16.67	2	33.33	9	2	2.46
12	Italy	6	703	117.17	0.58	3	50.00	2	33.33	19	1	2.46
13	Greece	5	649	129.80	0.65	2	40.00	0	0.00	3	4	2.05
14	India	4	620	155.00	0.77	1	25.00	0	0.00	1	3	1.64
15	Austria	4	613	153.25	0.76	2	50.00	1	25.00	10	1	1.64
16	Australia	4	475	118.75	0.59	2	50.00	1	25.00	10	3	1.64
17	Israel	3	836	278.67	1.39	1	33.33	0	0.00	1	5	1.23
18	Spain	3	520	173.33	0.86	1	33.33	1	33.33	9	2	1.23
19	Hong Kong	3	469	156.33	0.78	1	33.33	0	0.00	1	6	1.23
20	Sweden	3	468	156.00	0.78	1	33.33	0	0.00	2	7	1.23
21	Norway	3	424	141.33	0.70	2	66.67	1	33.33		5	1.23
	Total of 21 countries	284	50,545	177.98	0.89	62	21.83	54	19.01	47		
	Global total	244	48,981	200.74	1.00							

*TP* total papers, *TC* total citations, *CPP* citations per paper, *ICP* international collaborative papers, *RCI* relative citation index, *TLS* total link strength

**Fig. 1:**
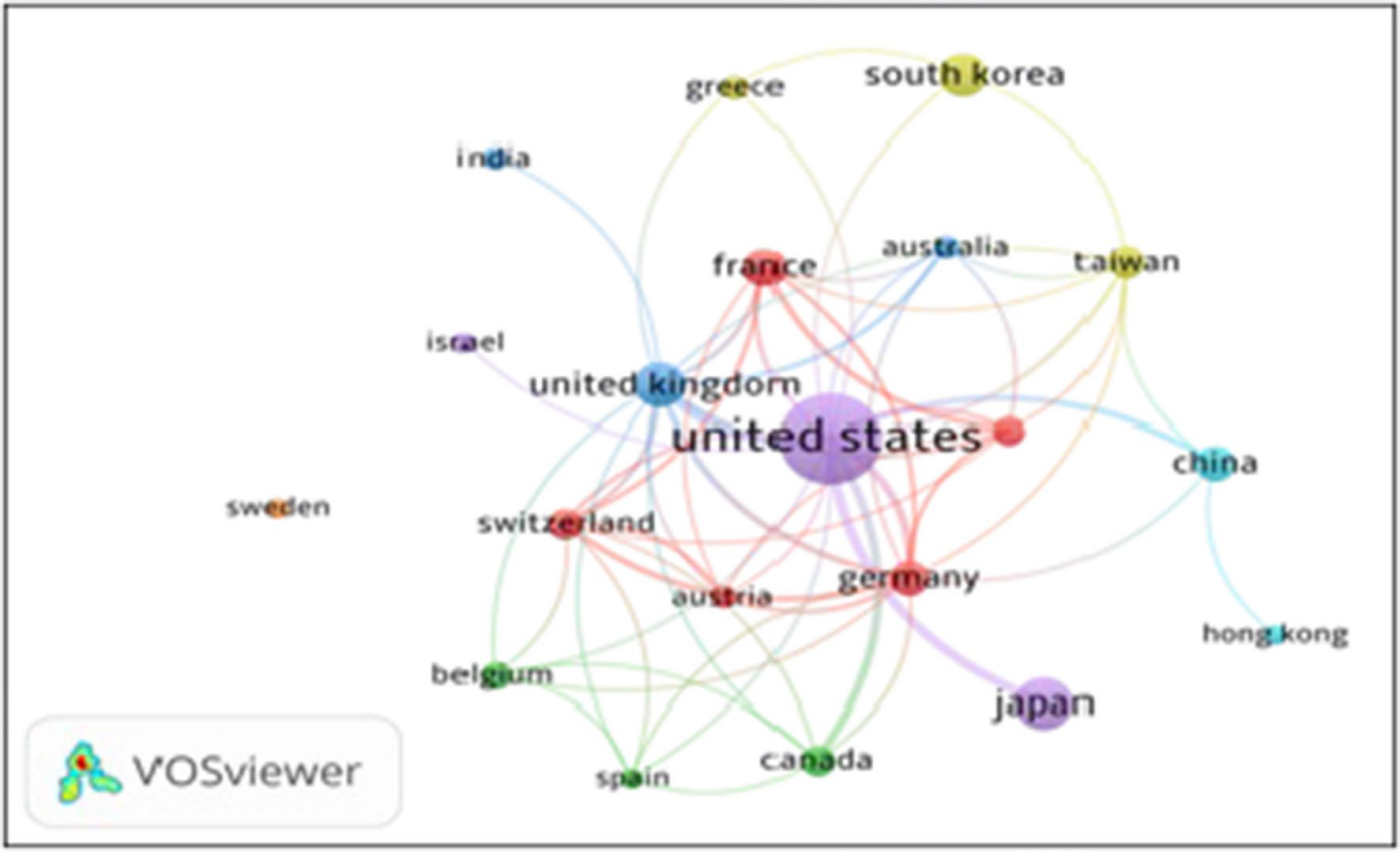
Bibliometric map based on the network of co-authorship relations among the top 21 countries *(with* > *2 papers)*. Seven different colored clusters represent 94 total link strengths: cluster 1 (France, Germany, Switzerland, Italy, and Austria), cluster 2 (Canada and Belgium), cluster 3 (India and Australia), cluster 4 (South Korea, Taiwan, and Greece), cluster 5 (USA, Japan, Israel, and Norway), cluster 6 (China and Hong Kong), and cluster 7 (Sweden)

The total link strength (TLS) of the top 21 countries varied from 1 to 47, with the highest links registered by USA (47), followed by the UK (32), and Germany (30). The individual country to country collaborative links varied from 1 to 6. The largest number of collaborative links was between USA and Canada (six), followed by USA and the UK (five), USA and Germany, USA and Japan, USA and the Netherlands, and the UK and the Netherlands (three each). The collaborative network linkage map of the top 21 countries with a minimum of 2 papers and 200 citations each ,using VOSviewer tool is shown in Fig. 1.

### Most productive and impactful organizations

There were 425 organizations that participated in 244 HCPs. Of these, the top 36 organizations contributed 3–12 papers each and together contributed 156 papers and 29,302 citations, accounting for 63.43% and 59.82%, respectively, of global papers and citations. Of the top 36 organizations, 18 were from the USA, 4 each from Japan and South Korea, and 3 from the UK. It was observed that only 12 organizations contributed above the group average productivity (4.33) of all 36 organizations. Only ten organizations registered CPP and RCI higher than the group average (187.83 and 0.94) of all 39 authors. Table 2 presents the bibliometric profile of the six most productive and six most impactful organizations.

**Table 2:** Bibliometric profile of the top six most productive and six most impactful organizations

No.	Organization name	TP	TC	CPP	RCI	ICP	%ICP
Top six most productive organizations							
1	Sinai Hospital of Baltimore, USA	12	2351	195.92	0.98	4	33.33
2	John Hopkins University, USA	11	3205	291.36	1.45	0	0.00
3	Harvard Medical School, USA	8	1340	167.50	0.83	2	25.00
4	Osaka University, Japan	8	1555	194.38	0.97	2	25.00
5	Hospital for Special Surgery, USA	7	1222	174.57	0.87	4	57.14
6	Good Samaritan Hospital, Baltimore, USA	6	1999	333.17	1.66	0	0.00
Top six most impactful organizations by citations per paper							
1	University of Arkansas for Medical Science, USA	3	1107	369	1.84	0	0
2	Good Samaritan Hospital, Baltimore, USA	6	1999	333.17	1.66	0	0
3	John Hopkins University, USA	11	3205	291.36	1.45	0	0
4	Hospital Henri Mondor, France	4	935	233.75	1.16	0	0
5	Nuffield Orthopedic Center, UK	3	666	222	1.11	0	0
6	Chang Gung Memorial Hospital, Taiwan	4	840	210	1.05	1	25
Top six most impactful organizations by total citations							
1	John Hopkins University, USA	11	3205	291.36	1.45	0	0
2	Sinai Hospital of Baltimore, USA	12	2351	195.92	0.98	4	33.33
3	Good Samaritan Hospital, Baltimore, USA	6	1999	333.17	1.66	0	0
4	Osaka University, Japan	8	1555	194.38	0.97	2	25
5	Harvard Medical School, USA	8	1340	167.5	0.83	2	25
6	Hospital for Special Surgery, USA	7	1222	174.57	0.87	4	57.14

*TP* total papers, *TC* total citations, *CPP* citations per paper, *ICP* international collaborative papers, *RCI* relative citation index, *TLS* total link strength

**Fig. 2:**
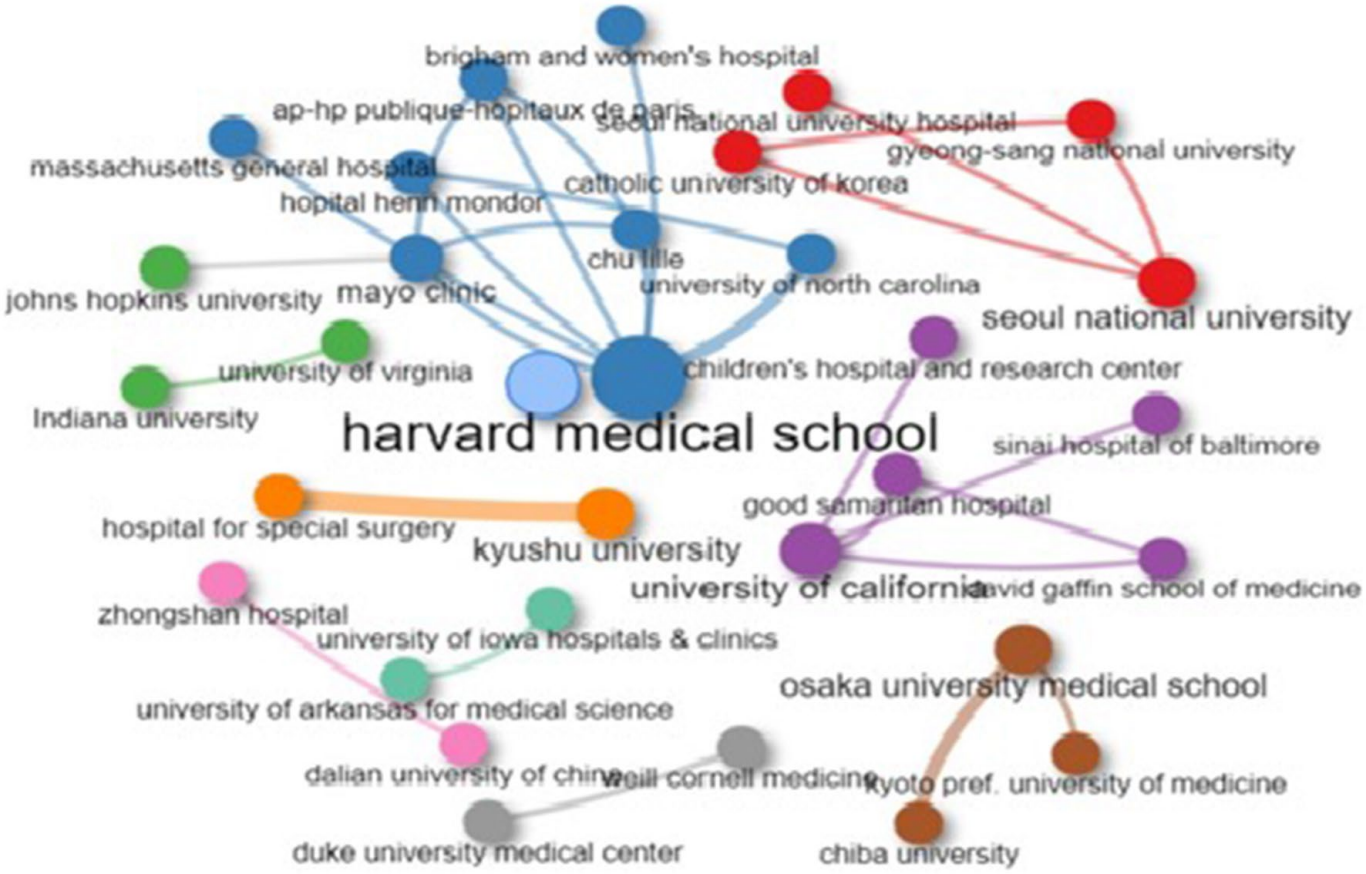
Bibliometric map based on the network of co-authorship relations among the top 36 organizations (with > 2 papers)

Among the top 36 organizations, only 15 organizations were involved in collaboration among themselves and their individual total collaborative link strength varied from 1 to 15, with the largest collaborative linkages in Harvard Medical School, USA (15 linkages), followed by John Hopkins University, USA and Seoul National University, South Korea (12 linkages each), and Sinai Hospital of Baltimore, USA (9 linkages). The largest number of collaborative linkages (four each) were between Sinai Hospital of Baltimore, USA and John Hopkins University, USA; Harvard Medical School, USA and Massachusetts General Hospital, USA; John Hopkins University, USA—C and Seoul National University and Seoul National University Bandung Hospital, South Korea. The collaborative linkage network map of the 36 top organizations is shown in Fig. 2 (prepared using the Biblioshiny software). Each node represents an organization, with the size of the node indicating the number of publications for an organization. The line thickness between the nodes is proportional to the number of publications in collaboration. The different colors represent different clusters with the same color nodes representing the same cluster. Among 36, all collaborative organizations are divided into 13 clusters. Cluster 1 consists of eight institutions, followed by cluster 2 of five institutions, cluster 3 of four organizations, followed by clusters 4 and 5 with three organizations each, clusters 6–10 composed of two organizations, and clusters 11–13 of individual organizations that are the top contributors.

### Most significant keywords

The keywords were extracted from the 2261 author keywords appearing in 244 HCPs using the text mining function of the VOSviewer software. Two keywords were said to co-occur if they both occur in the same bibliographic record. The distance between two keywords (two nodes) is approximately inversely proportional to the similarity (relatedness in terms co-occurrence) of the keywords. Hence, keywords with a higher rate of co-occurrence tend to be found closer to each other. The VOSviewer provides a clustering function, which assigns keywords to clusters based on their co-occurrence [14, 19]. Among 2261 author keywords appearing in 244 HCPs, 98 keywords were chosen as significant with frequency of occurrences varying from 10 to 1033, and they are listed in Table 3.

**Table 3:** The top 98 significant keywords (> 10 frequency of occurrence)

No.	Keyword (frequency)	TLS	Cluster	S. No.	Keyword (frequency)	TLS	Cluster
1	Femur head necrosis (174)	1033	1	50	Alendronic acid (10)	94	1
2	Femur head (110)	662	2	51	Fracture (10)	71	2
3	Total hip prosthesis (80)	586	2	52	Fracture nonunion (10)	68	3
4	Avascular necrosis (77)	538	3	53	Prosthesis design (9)	92	2
5	Bone necrosis (65)	429	1	54	Apoptosis (9)	78	1
6	Postoperative complications (44)	244	3	55	Osteoblast (9)	72	1
7	Surgical technique (42)	262	3	56	Sickle cell anemia (9)	49	1
8	Hip arthroplasty, replacement (32)	269	2	57	Bone cement (8)	75	2
9	Osteonecrosis (29)	215	1	58	Dexamethasone (8)	74	1
10	Hip osteoarthritis (28)	224	2	59	Fracture reduction (8)	66	3
11	Corticosteroid (26)	211	1	60	Hip surgery (8)	63	3
12	Prosthesis failure (25)	211	2	61	Diabetes mellitus (8)	60	1
13	Hip prosthesis (23)	216	2	62	Angiogenesis (8)	55	1
14	Osteoarthritis (23)	193	2	63	Fracture fixation (8)	53	3
15	Acetabulum (23)	178	2	64	Fracture healing (8)	50	3
16	Femur neck fracture (21)	167	3	65	Pathogenesis (7)	125	1
17	Hip arthroplasty (21)	161	2	66	Mesenchymal stem cells (7)	55	1
18	Decompression surgery (21)	134	4	67	Cell differentiation (7)	54	1
19	Rheumatoid arthritis (20)	169	2	68	Animal models (7)	51	1
20	Prognosis (20)	155	1	69	Preoperation evaluation (7)	51	2
21	Hip dislocation (20)	149	3	70	Perthe’s disease (7)	47	2
22	Hip joint (19)	167	2	71	Alcohol consumption (6)	586	1
23	Steroids (19)	140	1	72	Mesenchymal stem cells transplantation (6)	58	1
24	Osteotomy (19)	127	4	73	Orthopedic surgery (6)	48	3
25	Femur fracture (18)	116	3	74	Surgical decompression (6)	48	4
26	Bone graft (18)	107	4	75	Bone development (6)	45	1
27	Osteoarthritis, hip (17)	157	2	76	Femur intertrochanteric (5)	64	2
28	Glucocorticoids (17)	142	1	77	Vitamin D (5)	57	1
29	Hip disease (17)	114	2	78	Idiopathic disease (5)	54	1
30	Systemic lupus erythematous (16)	147	1	79	Azathioprine (5)	53	1
31	Osteolysis (16)	117	2	80	Autologous transplantation (5)	42	4
32	Prosthesis loosening (15)	136	2	81	Biomechanics (5)	41	2
33	Hip dysplasia (15)	117	2	82	Autologous bone marrow transplantation (5)	38	4
34	Bone marrow (15)	89	1	83	Femoroacetabular impingement (5)	37	2
35	Treatment outcome (14)	447	3	84	Immunohistochemistry (5)	36	2
36	Methylprednisolone (14)	115	1	85	Drug effect (5)	30	1
37	Corticosteroid therapy (14)	113	1	86	Hypertension (4)	53	1
38	Pathology (14)	110	2	87	Congenital hip dislocation (4)	46	2
39	Femur neck (12)	90	2	88	Protein expression (4)	38	1
40	Risk assessment (12)	80	3	89	Hyperbaric oxygen (4)	35	4
41	Osteosynthesis (12)	75	3	90	Extracorporeal lithotripsy (4)	33	4
42	Pathophysiology (11)	101	1	91	Blood clotting disorders (3)	31	1
43	Bone transplantation (11)	82	4	92	Fibrinolysis (3)	28	1
44	Osteoporosis (11)	81	1	93	Gaucher disease (3)	24	1
45	Weight bearing (11)	81	3	94	Thrombophilia (3)	23	1
46	Hip fracture (11)	78	3	95	Bone regeneration (3)	15	1
47	Conservative treatment (11)	72	4	96	Kidney transplantation (2)	28	1
48	Biophosphonic acid derivatives (10)	116	1	97	Acetabulum fracture (2)	26	3
49	Prednisone (10)	106	1	98	Radiation injuries (2)	10	2

*TLS* Total link strength

**Fig. 3:**
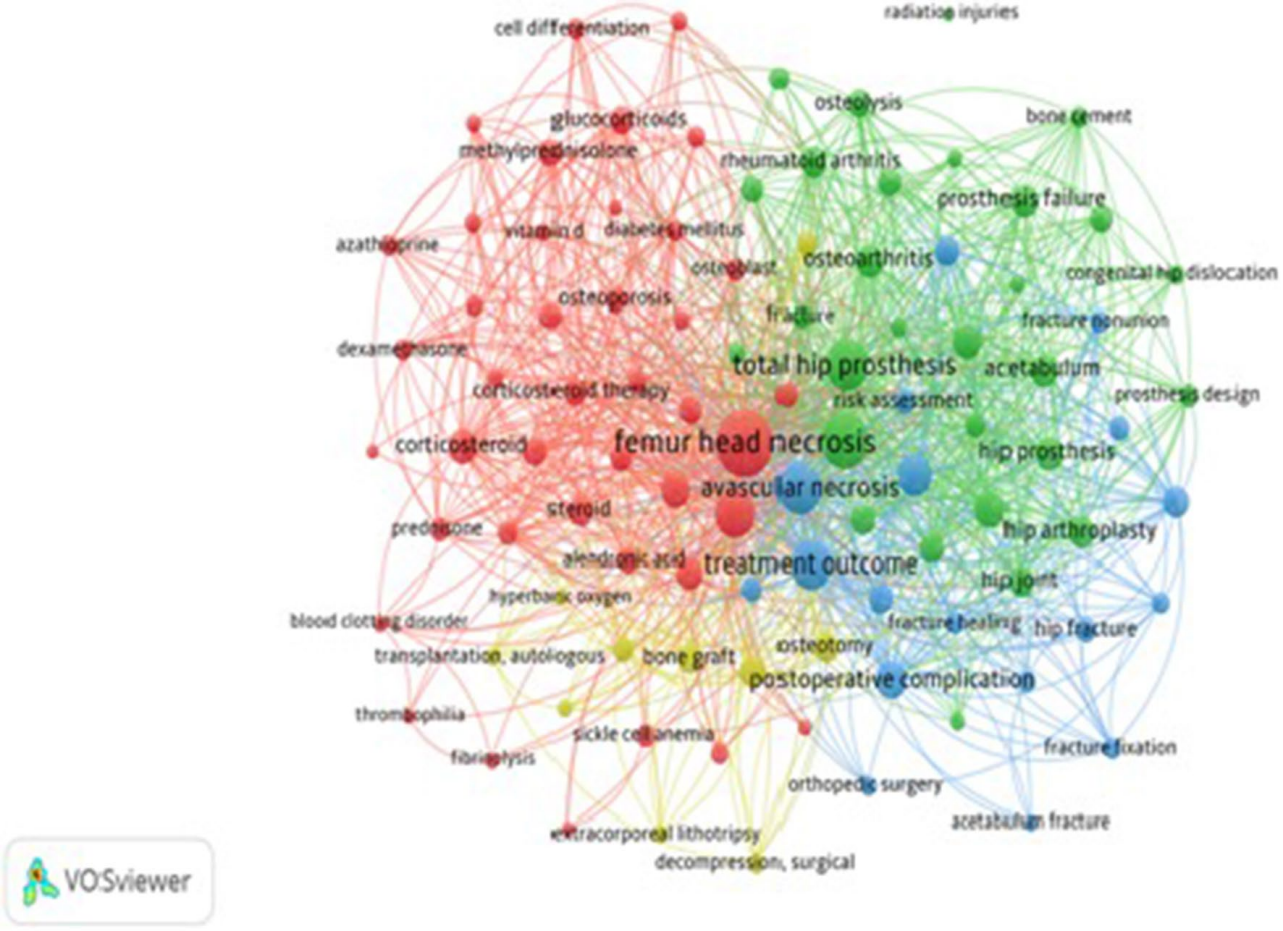
Co-occurrence map of 98 significant keywords

The co-occurrence of 98 selected keywords indicates the important areas of research in AVNFH research and are shown in Fig. 3, by different colors. Cluster 1 (red) has 41 keywords, cluster 2 (green) has 29 keywords, cluster 3 (blue) has 18 keywords, and cluster 4 (yellow) includes 10 keywords.

### Most productive and impactful authors

There were 1625 authors who participated in 244 HCPs. Of these, the top 38 authors contributed 3–18 papers each and together contributed 167 papers and 33,302 citations, accounting for 68.44% and 67.99%, respectively, in global papers and citations. Of the top 38 authors, most were from the USA (14), followed by Japan (11) and South Korea (5). The bibliometric profile of the top six most productive and six most impactful authors is presented in Table 4. Only nine authors contributed above the group average productivity (4.39) of all 38 authors. Only 12 authors registered CPP and RCI higher than the group average (199.41 and 0.99) of all 38 authors.

**Table 4:** Bibliometric profile of the top six most productive and six most impactful journals

No.	Name of the author	Affiliation of the author	TP	TC	CPP	RCI	ICP	% ICP
Top six most productive authors								
1	R.A. Mont	Sinai Hospital of Baltimore, USA	18	4361	242.28	1.21	0	0.00
2	K.H. Koo	Seoul National University Bundang Hospital, S. Korea	11	1432	130.18	0.65	1	9.09
3	N. Sugano	Osaka University, Japan	9	1552	172.44	0.86	0	0.00
4	D.S. Hungerford	John Hopkins University, USA	8	2334	291.75	1.45	0	0.00
5	L.C. Jones	John Hopkins University, USA	7	1459	208.43	1.04	0	0.00
6	J.R. Urbaniak	Duke University Medical Center, USA	5	1063	212.60	1.06	0	0.00
Top six most impactful authors by citations per paper								
1	R. Ganj	University of Bern, Switzerland	5	2012	402.4	2	2	40
2	R.S. Weinstein	University of Arkansas for Medical Science, USA	3	1107	369.0	1.84	0	0
3	M.E. Steinberg	University of Pennsylvania, USA	3	885	295.0	1.47	0	0
4	D.S. Hungerford	John Hopkins University, USA	8	2334	291.75	1.45	0	0
5	P. Hernigou	Hospital Henri Mondor, France	3	817	272.33	1.36	0	0
6	R.A. Mont	Sinai Hospital of Baltimore, USA	18	4361	242.28	1.21	0	0
Top six most impactful authors by total citations								
1	R.A. Mont	Sinai Hospital of Baltimore, USA	18	4361	242.28	1.21	0	0
2	D.S. Hungerford	John Hopkins University, USA	8	2334	291.75	1.45	0	0
3	R. Ganj	University of Bern, Switzerland	5	2012	402.4	2	2	40
4	N. Sugano	Osaka University, Japan	9	1552	172.44	0.86	0	0
5	L.C. Jones	John Hopkins University, USA	7	1459	208.43	1.04	0	0
6	K.H. Koo	Seoul National University Bundang Hospital, S. Korea	11	1432	130.18	0.65	1	9.09

*TP* total papers, *TC* total citations, *CPP* citations per paper, *ICP* international collaborative papers

Among the top 38 authors, only 21 authors collaborated among themselves, and their individual total collaborative linkages varied from 1 to 34, with the largest collaborative linkages by R.A. Mont (34), followed by N. Sugano (19) and D.S. Hungerford and D.R. Marker (14 each). The bilateral collaborative linkages among 21 authors varied from 1 to 6, with the largest number of linkages between R.A. Mont and L.C. Jones (six), followed by R.A. Mont and D.R. Marker (five), R.A. Mont and T.N. Seylor, R.A. Mont and T.M. Seyler, L.C. Jones and D.S Hungerford, and K. Ohzono and K. Takaoka (four each).

**Fig. 4:**
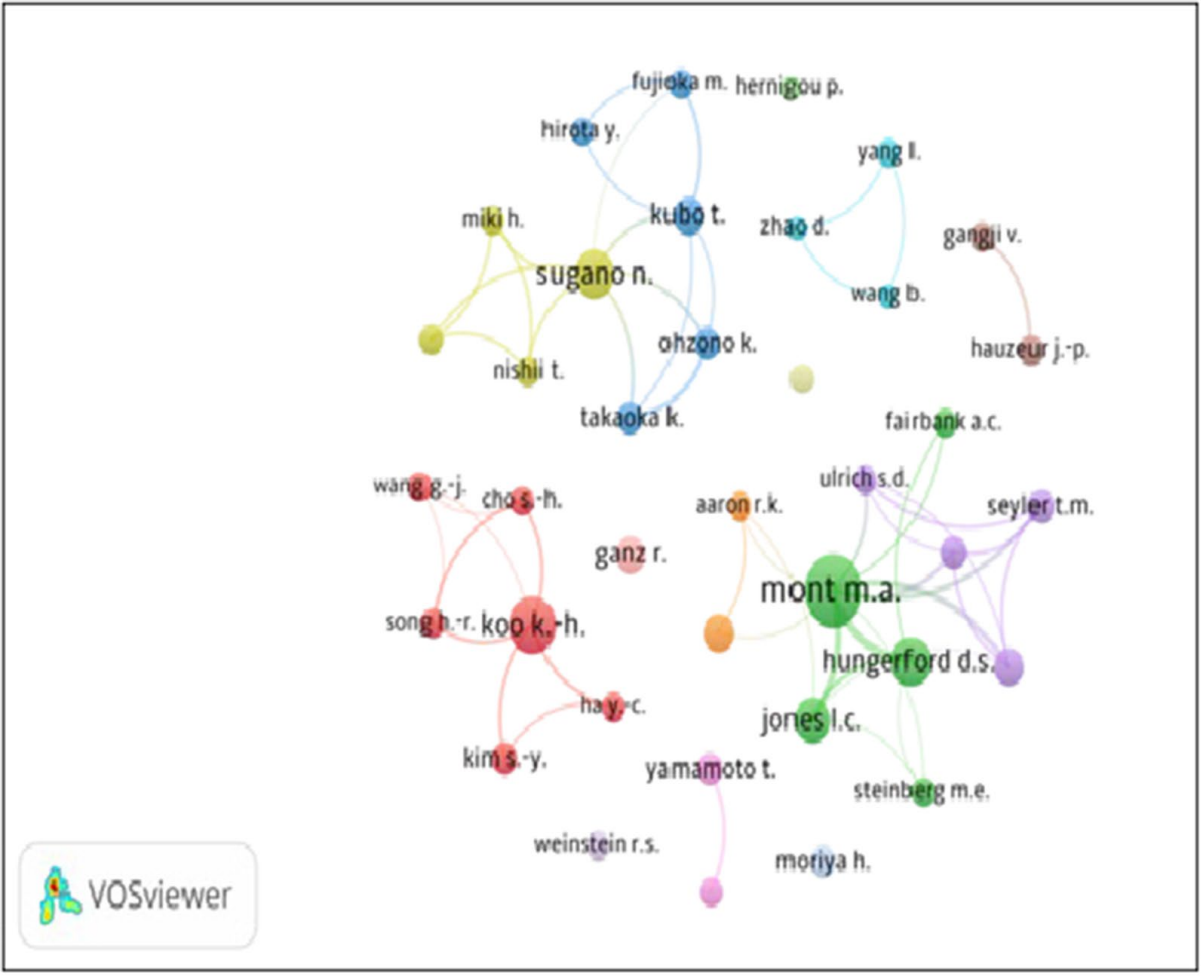
Bibliometric map based on the network of co-authorship relations among the top 38 authors (with > 2 papers)

The collaborative linkages network map of the 38 top authors (with more than two papers) is depicted in Fig. 4 (prepared using the Biblioshiny software). Each node represents an organization, with the size of the node indicating the number of publications for an organization. The line thickness between the nodes is proportional to the number of publications in the collaboration. The different colors represent different clusters, with the same color nodes representing the same cluster. All 38 authors are presented in 14 clusters with 137 total linksh. Cluster 1 had six authors, followed by clusters 2 and 3 with five authors each, clusters 4 and 5 with four authors each, cluster 6 with three authors, clusters 7–9 with two authors each, and clusters 10–14 with one author each.

### Most productive and impactful journals

Of the 84 journals appearing in 244 HCPs, the top 24 have published 2–50 papers and together published 181 papers, registering 32,970 citations, accounting for 74.18% and 67.31 of global papers and citations, respectively. Table 5 presents the bibliometric profile of top six most productive and most impactful journals.

**Table 5:** Bibliometric profile of the top six most productive and six most impactful journals

No.	Name of the source	TP	TC	CPP	%TP
Top six most productive journals					
1	Journal of Bone and Joint Surgery	50	9550	191.00	20.49
2	Clinical Orthopedics and Related Research	36	6024	167.33	14.75
3	Journal of Bone and Joint Surgery—Series B	35	7378	210.80	14.34
4	Journal of Arthroplasty	9	1205	133.89	3.69
5	Arthritis and Rheumatism	4	589	147.25	1.64
6	Radiology	4	515	128.75	1.64
Top six most impactful journals by citations per paper					
1	Seminars in Arthritis and Rheumatism	2	756	378.00	0.82
2	New England Journal of Medicine	3	912	304.00	1.23
3	Journal of Clinical Endocrinology and Metabolism	2	492	246.00	0.82
4	Rheumatology	2	433	216.50	0.82
5	Journal of Bone and Joint Surgery—Series B	35	7378	210.80	14.34
6	Journal of Orthopaedic Science	2	412	206.00	0.82
Top six most impactful journals by total citations					
1	Journal of Bone and Joint Surgery	50	9550	191.00	20.49
2	Journal of Bone and Joint Surgery—Series B	35	7378	210.80	14.34
3	Clinical Orthopedics and Related Research	36	6024	167.33	14.75
4	Journal of Arthroplasty	9	1205	133.89	3.69
5	New England Journal of Medicine	3	912	304.00	1.23
6	Seminars in Arthritis and Rheumatism	2	756	378.00	0.82

*TP* total papers, *TC* total citations, *CPP* citations per paper

**Fig. 5:**
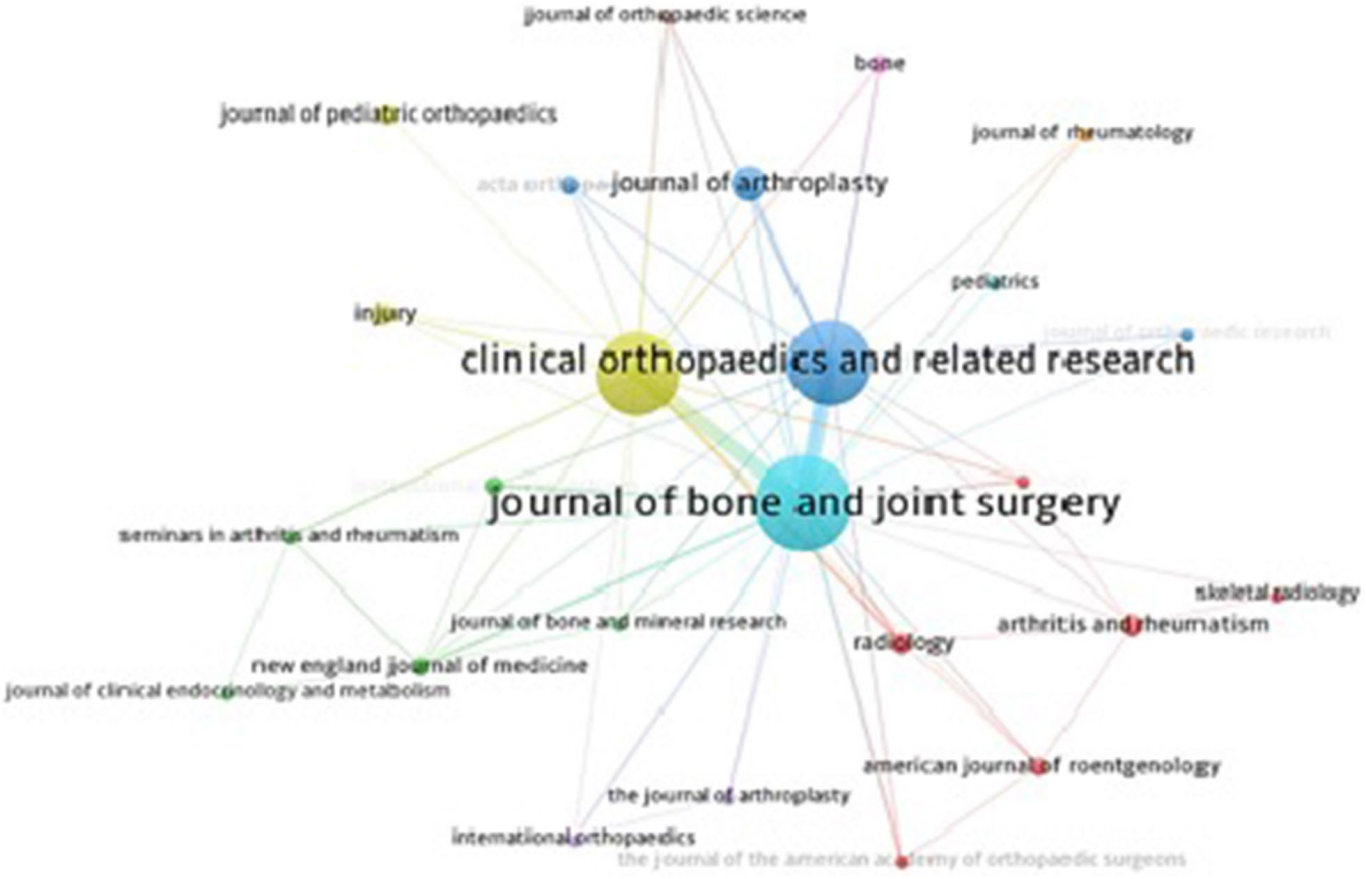
Co-citation network visualization of top 25 most productive journals

A co-citation network map of the top 25 most productive journals is shown in Fig. 5, which depicts these journals in nine clusters with 309 link strengths. In the map, two or more journals that cover closely related topics are placed close to one another, and those covering fundamentally different topics are located far from each other. The circle and font size of a journal node are proportional to the frequency of its co-citations. Cluster 1 (red) has six journals, cluster 2 (green) has four journals, cluster 3 (blue) has four journals, cluster 4 (yellow) includes three journals, clusters 5 and 6 have two journals each), and clusters 7–9 have one journal each.

### Top highly cited papers

**Fig. 6:**
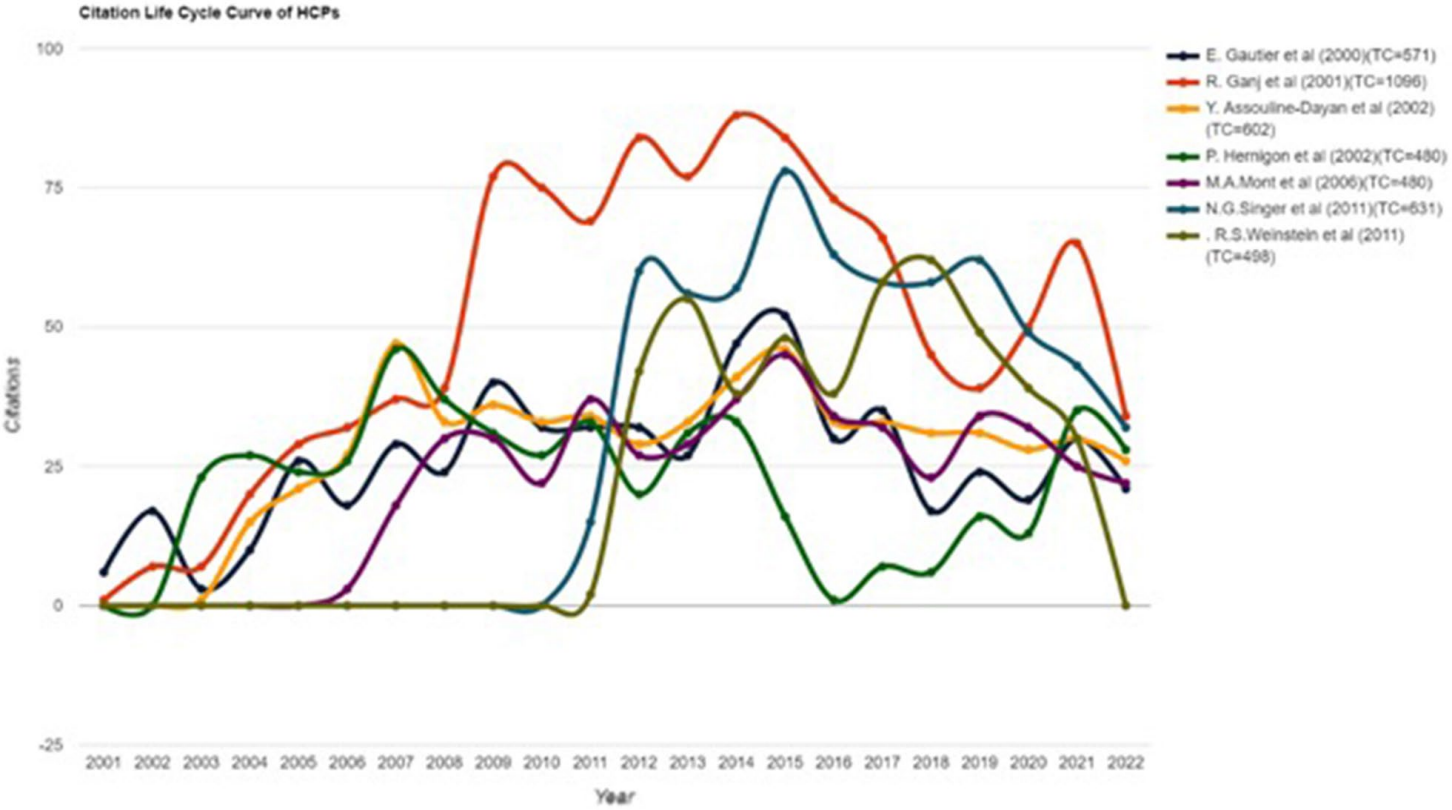
The citation life cycle of the highly cited papers

Citations are commonly taken as input for several influential evaluative metrics used to assess researchers’ performance. Nevertheless, little effort has been devoted to understanding and quantifying how article citations evolve over the years following an article’s publication [20]. It was observed that not all HCPs have the same citation life cycle curves, i.e., curves of frequency of citations received versus time. All HCPs have a citation life extending over the entire period studied. It was also observed that most of these articles were still being cited 12–25 years after they had been published. Normally, in citation lifetime cycles, in the first years after publication, articles generally receive a small but growing number of citations until, and then eventually they reach a peak from which they then decline. There are pronounced differences in the life cycle curves of the HCPs studied here, and they are classified as three distinct types as described below (Table 6 and Fig. 6).

**Table 6:** Number and ratio of citations to total citations received within 2 and 5 years of the nine most highly cited papers

No.	Authors	Year of publication	TC	TC (after 2 years)	TC (after 5 years)	Peak citations	Average citations per year (citation span period)
Paper 2	M.A. Mont et al.	1995	931	29 (3.11%)	94 (10.10%)	60 citations (after 10 years)	34.48 (27 years)
Paper 6	M.E. Steinberg et al.	1995	561	12 (2.14%)	50 (8.91%)	35 citations (after 10 years)	20.78 (27 years)
Paper 5	E. Gautier et al.	2000	571	23 (4.03%)	62 (10.85%)	52 citations (after 14 years)	25.95 (22 years)
Paper 1	R. Ganj et al.	2001	1096	15 (1.37%)	96 (8.76%)	88 citations (after 13 years)	49.20 (21 years)
Paper 4	Y. Assouline-Dayan et al.	2002	602	16 (2.66%)	111 (18.44%)	47 citations (after 4 years)	30.10 (20 years)
Paper 9	P. Hernigon et al.	2002	480	8 (1.67%)	43 (8.96%)	46 citations (after 11 years)	24.0 (20 years)
Paper 8	M.A. Mont et al.	2006	480	51 (10.63%)	140 (29.17%)	45 citations (after 8 years)	30.0 (16 years)
Paper 3	N.G. Singer et al.	2011	631	131 (20.76%)	329 (52.14%)	78 citations (after 3 years)	57.36 (11 years)
Paper 7	R.S. Weinstein et al.	2011	498	99 (19.88%)	223 (44.78%)	62 citations (after 6 years)	49.8 (11 years)

## Discussion

Bibliometrics is a science that uses statistical and mathematical procedures for the statistical evaluation of publications, which allow us to assess the impact, research performance, and author productivity. Bibliometrics has garnered major interest in recent years as it shows the publication trends, knowledge evolution, and evidence-based practice throughout the years. The quantitative analysis of a field has been gauged using reviews, meta-analyses, and bibliometric studies [10–14]. Reviews and meta-analyses have their own limitations and advantages, but bibliometric studies play a much more important role to identify research characteristics of a field. Under the rubric of bibliometrics, citation counts have been incorporated into metrics intended to measure the impact of researchers, papers, journals, universities, and even countries. Of late, many countries are moving toward research policies that emphasize excellence; consequently, they develop evaluation systems to identify universities, research groups, and individual researchers that can be said to be “excellent.” Such excellence could be measured by citation counts [11–14]. As the subject of research excellence has received increasing attention (in science policy) over the last few decades, increasing numbers of bibliometric studies have been published, dealing with, characterizing, and ranking highly cited papers in different disciplines [14]. In addition, articles with high citations are considered central to research. Therefore, HCPs provide evidence and information about research trends and scientific progress in a specific field. The characteristics of HCPs help us to identify important advances and their scientific impact. However, criticisms to this approach have been raised. Given the intrinsic nature of the scientometric analysis, which mainly focuses on the rate of citation, this could be not strictly related to the quality. Indeed, in some instances, simply describing a concept before others was enough to be highly cited. This represents a limitation of the present approach, which cannot be “measured” in a scientometric analysis. Therefore, results from the present study, and scientometric analysis in general, must be interpreted with caution.

AVNFH-related scientific publications have increased during the last 30 years and reached 8496 at 30 September 2022. However, a few bibliometric studies exist on this topic, using mainly Web of Science (WOS) databases. These bibliometric studies [15–17]. examine the quantitative and qualitative aspects of research activity, identify important actors (countries, institutions, authors, and journals) in research, and map knowledge in this field using important keywords. A bibliometric study [21] has identified the top 100 HCPs (with more than 30 citations) with a view to analyze their characteristics and influence of research, using WOS up to 27 January 2021. This study has many limitations in terms of limited coverage of HCPs (only top 100) and covered HCPs with more than 30 citations only.

We noticed that only 2.87% of a total of 8496 papers were HCPs with 100 or more citations, and received a total of 200.78 CPP. Collaborative research papers, with one other organization, received a higher CPP of 245.39, than with many organizations (average CPP of 165.67). However, the international collaborations yielded a higher CPP of 172.54 than the national collaborations (CPP of 151.03). Only 12.3% of HCPs were involved in international collaboration, and the USA was the center of dominating bilateral and multilateral collaboration with the maximum number of countries. Comparatively strong collaborations existed between organizations within the same country and moderate collaboration was observed between organizations across major participating countries. Japan, the UK, South Korea, and Germany have shown moderate collaborative linkages with other countries. To increase the research output and raise information impact, there is a need to increase international collaboration linkages among various countries.

Of the 36 organizations contributing two or more HCPs in AVNFH, USA was the top with 18 papers, followed by Japan and South Korea (four each), the UK (three), and China and France (two each), reflecting the dominance of these countries in research output. Not only did these organizations contribute the maximum HCPs but they also created the largest impact in this field.

Of the 38 authors contributing 2 or more HCPs in this field, 14 came from the USA, 11 were from Japan, 5 from South Korea, and 4 from China. R.A. Mont (USA) contributed the largest number of papers (18), followed by K.H. Koo (South Korea) (11 papers), and N. Sugano (Japan)(9 papers). However, R. Ganz (402.40 and 2.0), R.S. Weinstein (369.0 and 1.84), M.E. Steinberg (295.0 and 1.47), and D.S. Hungerford (291.75 and 1.45) made the largest impact in terms of CPP and RCI, respectively. Sixty percent of these top authors were involved in collaboration with fellow colleagues. It is to be noted that the major collaboration was seen between the authors from the same organizations or among authors from two organizations in the same countries. However, few collaborative linkages were observed between authors across countries.

External funding was received in 11.9% of HCPs and the major funding agencies were from the USA, Japan, and South Korea. The leading funding agencies were National Institute of Health, USA (12 papers), National Institute of Arthritis & Musculoskeletal & Skin Diseases, USA, and the US Department of Health & Human Service (six papers each). The overall share of external funding in country output among top 20 countries was 19.0%, with 54.6% share in China, followed by Canada (37.5%), and Germany (36.7%). We believe that there is an urgent need to support and enhance external funding in this area by national funding agencies, so that better research outcomes can be expected from the research results. The citations impact (CPP and RCI, respectively) was maximum for the authors from Switzerland (316.75 and 1.58), Israel (278.67 and 1.39), the UK (195.14 and 0.97), and USA (191.47 and 0.95). The contribution from developing countries was very small with only four papers from India and one paper each from Brazil and Iran. Hence, more research is needed from these countries with large populations.

The majority of HCPs (71.7%) were original articles, followed by review articles (15.2%), and involved human subject in 98.4%. Clinical studies accounted for the largest number of papers, followed by treatment and risk factors.

In all 2261 author keywords appearing in 244 HCPs, 98 keywords were significant, as measured by their higher frequency of occurrences. The co-occurrences analysis of these significant keywords provides the broad ideas about the prominent subjects, which are depicted in their cluster analysis. Four prominent clusters were observed pointing towards the priorities assigned to research in this area and can help the researchers to gauge the emerging areas of research in this field.

It was observed that not all highly cited papers have the same citation life cycle curves, i.e., curves of frequency of citations received versus time [20]. All of the nine HCPs have a citation life extending over the entire period studied. It was also observed that most of these articles were still being cited 12–25 years after they were published. Normally in a citation lifetime cycle, in the first years after publication, articles generally receive a small but growing number of citations, until eventually they reach a peak from which they then decline [20].

Considering the clinical significance of AVNFH research and the importance of HCPs, this detailed bibliometric analysis explored the citations, authors, journals, publishing countries, and keyword information. It also visualized the relationships between the most frequently occurring concepts and keywords and collaboration linkages among participating countries, organizations, and authors. This study also provides research insights into current developments in AVNFH in the last 30 years. We believe that it will help the researchers to understand the research influence and trends and provide reference for future research.

## Limitations

Despite the rigorous bibliometric analysis made in this study, there were a few shortcomings and limitations. For example, we only analyzed bibliometric data from the Scopus database, which may have missed other relevant publications covered in other databases such as Web of Science, etc. In addition, we had made an analysis of highly cited papers, which only includes core literature in a field and, as a result, missed out on concepts from allied literature.

## Conclusions

A small percentage (2.9%) of the 8496 publications on AVNFH received 100 or more citations in the last three decades. The 244 HCPs together received 48,981 citations, averaging 200.78 citations per paper. Only 11.9% of HCPs received external funding and there is an urgent need to support and enhance external funding in this area by national funding agencies. To increase the research output and raise information impact, there is a need to increase international collaboration linkages among various countries. The contribution from developing countries was very small and more research is needed from these countries with large populations.

## Data Availability

Supplementary raw data can be obtained after reasonable request to the corresponding author.

## Abbreviations

AVNFH: Avascular necrosis of the femoral head
HCS: Highly cited studies
CPS: Citations per study
TS: Total studies
TC: Total citations
ICP: International collaborative studies
RCI: Relative citation index
TLS: Total link strength
